# Effect of the pregnant mice with Graves’ disease on thyroid function in the offspring

**DOI:** 10.1186/1687-9856-2013-S1-P144

**Published:** 2013-10-03

**Authors:** Qianqi Liu, Yue Yu, Shu Wang, Chunrong Chen, Xing Shi, Shining Ni, Wei Gu, Ziyang Zhu

**Affiliations:** 1 Department of Endocrinology, Nanjing Children’s Hospital Affiliated to Nanjing Medical University, Nanjing, Jiangsu Province, P.R.China 210008; 2 Department of Endocrinology, Ruijin Hospital Affiliated to School of Medicine, Shanghai Jiaotong University, Shanghai 200025; 3 Cedars-Sinai Research Institute and School of Medicine, UCLA, California 90048, USA

**Keywords:** Graves' disease, animal model, thyroid autoantibodies, breast milk, offspring, thyroid function

## Objective

1. To prepare an animal model by immunizing female Balb/c mice with Adenovirus Expressing TSHR A-subunit(Ad-TSHR-289).

2. To measure autoantibodies and thyroxine in all offsprings' serum and their mothers' breast milk.

3. To investgate the effect of the pregnant mice with Graves’ disease on thyroid function in the offspring.

## Methods

1. To amplify the Adenovirus Expressing TSHR A-subunit(Ad-TSHR-289) and the control adenovirus (Ad-β-gal);

2. Female Balb/c mice aged 6 weeks were divided randomly into the experimental group and the control group, then injected intramuscularly with Ad-TSHR-289 (3.3×10^8^PFU/50μlPBS) and the control adenovirus(Ad-β-gal 3.3×10^8^ PFU /50μlPBS) three times at 3 weeks intervals.

3. In 1 wk after the second injection, the levels of T4 were measured. All mice were sacrificed 4 wk after the last immunization, and then Thyroxine(T4), as well as TSH-receptor antibodies(TRAb) and the morphology of thyroid, were then examined.

4. When the immunization was over, both of groups were mated with the male mice. The experimental group were divided into three groups again. They were sacrificed at 1 d, 1 wk or 3 wk after delivery. At the same time, the breast milk from the infants' stomach were collected. T4, as well as TRAb, Thyroid peroxidase antibodies(TPOAb), Thyroglobulin antibodies(TGAb) in serum and the morphology of thyroid, were be examined.

5. T4 were tested by radio immunoassay. TRAb, TPOAb, TGAb were tested by electrochemiluminescent immunoassay.

6. Their thyroids were removed for histological examination.

## Results

### Part 1

1. Purified recombinant adenovirus with the target gene-TSHR-289 was amplified, which size is about 800bps.

2. 7 mice in experiment group were suffered with Graves' hyperthyroidism. The incidence rate was 70%. The levels of serum T4 and TRAb in the experimental group were significantly higher than those of the control(P<0.05).

### Part 2

1. The levels of serum T4 and TRAb of mothers at 1d, 1wk and 3wk after delivery in the experimental group were significantly higher than those of the control(P<0.05). 22, 18 and 23 mothers after 1d,1wk and 3wk delivery in the experimental group were suffered with Graves' hyperthyroidism respectively. The incidence rate respectively were 73.3%, 60% and 76.67%.

2. TRAb levels could be tested in breast milk from 9 mice at 1d after delivery, TPOAb levels >0.5IU/L in breastmilk from 15 mice, and TGAb in breast milk from 4 mice. At 1wk after delivery, TRAb levels could be tested in breast milk from 4 mice, TPOAb in breast milk from7 mice , and TGAb in breast milk from 1 mice. TRAb can not be tested in mice' breast milk in 3wk after delivery, 2 mice could be tested TPOAb from their breast milk, and 3 mice could be tested TGAb from their breast milk. The levels of TPOAb in the breast milk after 1d delivery in the experiment group were significantly higher than those of the control group(P<0.05).

3. 114, 129 offspring 1wk and 3wks-old in the experiment group were suffered with Graves' hyperthyroidism. The incidence rate respectively were 63.3% and 71.7%. The levels of serum T4, TRAb, TPOAb and TGAb in offspring 1wk and 3wks-old in the experimental group were significantly higher than those of the control(P<0.05).

## Conclusions

1. An animal model of Graves' disease is successfully prepared by immunization with Ad-TSHR-289.

2. The higher levels of thyroid autoantibodies could result in neonatal thyroid disease, whose mothers were suffered with Graves’ disease.

3. The levels of thyroid autoantibodies in the mother with Graves’ disease could affect the offspring's thyroid function through the placenta and breast milk.

